# Computational Contact Pressure Prediction of CoCrMo, SS 316L and Ti6Al4V Femoral Head against UHMWPE Acetabular Cup under Gait Cycle

**DOI:** 10.3390/jfb13020064

**Published:** 2022-05-23

**Authors:** J. Jamari, Muhammad Imam Ammarullah, Gatot Santoso, S. Sugiharto, Toto Supriyono, Akbar Teguh Prakoso, Hasan Basri, Emile van der Heide

**Affiliations:** 1 Department of Mechanical Engineering, Faculty of Engineering, Diponegoro University, Semarang 50275, Central Java, Indonesia; j.jamari@gmail.com; 2 Undip Biomechanics Engineering & Research Centre (UBM-ERC), Diponegoro University, Semarang 50275, Central Java, Indonesia; 3 Department of Mechanical Engineering, Faculty of Engineering, Pasundan University, Bandung 40264, West Java, Indonesia; gatot.santoso@unpas.ac.id (G.S.); sugih.sugiharto@unpas.ac.id (S.S.); supriyono.toto@unpas.ac.id (T.S.); 4 Department of Mechanical Engineering, Faculty of Engineering, Sriwijaya University, Indralaya 30662, South Sumatra, Indonesia; prakoso@unsri.ac.id (A.T.P.); hasan_basri@unsri.ac.id (H.B.); 5 Laboratory for Surface Technology and Tribology, Faculty of Engineering Technology, University of Twente, P.O. Box 217, 7500 AE Enschede, The Netherlands; e.vanderheide@utwente.nl

**Keywords:** CoCrMo, contact pressure, SS 316L, Ti6Al4V, total hip arthroplasty, UHMWPE

## Abstract

Due to various concerns about the use of metal-on-metal that is detrimental to users, the use of metal as acetabular cup material was later changed to ultra high molecular weight polyethylene (UHMWPE). However, the wear on UHMWPE releases polyethylene wear particles, which can trigger a negative body response and contribute to osteolysis. For reducing the wear of polyethylene, one of the efforts is to investigate the selection of metal materials. Cobalt chromium molybdenum (CoCrMo), stainless steel 316L (SS 316L), and titanium alloy (Ti6Al4V) are the frequently employed materials. The computational evaluation of contact pressure was carried out using a two-dimensional axisymmetric model for UHMWPE acetabular cup paired with metal femoral head under gait cycle in this study. The results show Ti6Al4V-on-UHMWPE is able to reduce cumulative contact pressure compared to SS 316L-on-UHMWPE and CoCrMo-on-UHMWPE. Compared to Ti6Al4V-on-UHMWPE at peak loading, the difference in cumulative contact pressure to respective maximum contact pressure is 9.740% for SS 316L-on-UHMWPE and 11.038% for CoCrMo-on-UHMWPE.

## 1. Introduction

The use of metal-on-metal bearing was previously the surgeon’s choice to perform a total hip joint replacement surgery, especially in several developing countries such as Indonesia. According to the EU—Indonesia Business Network [1], Indonesia still has to import more than 90% of medical devices, including total hip prostheses. The metal-on-metal bearing can meet the needs of the local market without having to import from outside parties. This is due to these bearings using local materials that are easily available, the ease of the fabrication process, and the relatively affordable cost compared to other bearings. Unfortunately, several complications from metal-on-metal cause considerations that require choosing another bearing for total hip prosthesis [2]. This is also supported by the statement of the Australian Orthopaedic Association (AOA) [3], which explains the case of metal-on-metal failure is relatively high compared to the other bearing options available in the market today.

To deal with this complication considering the condition of developing countries such as Indonesia, the use of an acetabular cup with polyethylene material to replace a metal acetabular cup as a counterpart of a metal femoral head is a rational option [4]. Replacing the metal acetabular cup material with polyethylene can reduce the negative effects caused by metal-on-metal, such as tissue constraints in the body [5], aseptic loosening [6], and bone loss due to the release of metal ions [7]. In addition, polyethylene material is also a material that is relatively cheap and easy to produce compared to ceramic. Also, ceramics are brittle and sound squeaky, which is the rationale for not choosing this material [8].

One type of polyethylene that is widely used for bearings of a total hip prosthesis is ultra high molecular weight polyethylene (UHMWPE) [9,10]. However, there are concerns of negative biologic responses for implant users due to polyethylene particles that lead to osteolysis. In the combination of a metal femoral head and a UHMWPE acetabular cup, the wear of polyethylene can be minimized by selecting the right metal material for the femoral head. This is important considering that the longevity of a total hip prosthesis can be achieved by minimizing the wear of its components. Several metal materials available in Indonesia can be used, including cobalt chromium molybdenum (CoCrMo) [11], stainless steel 316L (SS 316L) [12], and titanium alloy (Ti6Al4V) [13].

Preclinical studies evaluating computational wear using the finite element method are crucial in predicting long-term wear of postoperative hip implants with a relatively short time required [14,15,16]. Contact pressure is one aspect that affects wear, so it is necessary to study the contact pressure on implant bearings because contact pressure and wear have a relationship based on the Archard wear equation [17]. The results of this investigation are also useful for a surgeon’s referral in carrying out surgical operations or minimizing experimental and clinical investigations that take a longer time rather than computational investigation [18].

Previous studies of contact pressure on metallic bearings of hip implants have been carried out by Wang et al. [19] by examining the correlation between acetabular cup orientation with a range of motion and contact pressure in metal-on-metal hip resurfacing prosthesis. Furthermore, [20] Mattei and Puccio investigated the effect of friction on bearings against wear in a metal-on-metal total hip prosthesis. Next, Shankar and Nithyaprakash [21] carried out computational simulations of contact pressure on a hard-on-soft total hip prosthesis by studying Al_2_O_3_-on-UHMWPE, CoCrMo-on-UHMWPE, and ZrO_2_-on-UHMWPE bearings. Based on previous research, computational evaluation of the contact pressure on a metallic bearing of a hip joint prosthesis is mostly done for metal-on-metal, and it is still rare for research focusing on metal-on-UHMWPE to investigate the choice of metal material to reduce contact pressure that is useful as a preliminary study before evaluating wear. There are many previous study found, not including nonlinear plastic characteristics of UHMWPE modelling, which could affect the computational simulation results. Bearing studies on total hip arthroplasty have focused on European hip joint geometry and material selection oriented towards leading countries. Unfortunately, research on Indonesian hip joint geometry (mostly used by Asians) and material selection oriented towards developing countries is difficult to find.

The main aim of the current investigation is to minimize contact pressure in the metal-on-UHMWPE bearing of a total hip prosthesis by examining different metal femoral head materials under gait cycle. The plastic nonlinearity of UHMWPE was taken into account in the present work. Two-dimensional axisymmetric finite element analysis to simulate metal-on-UHMWPE bearing based on Indonesian hip joint geometric size was carried out to accommodate the evaluation of the contact pressure.

## 2. Materials and Methods

### 2.1. Finite Element Model

**Figure 1:**
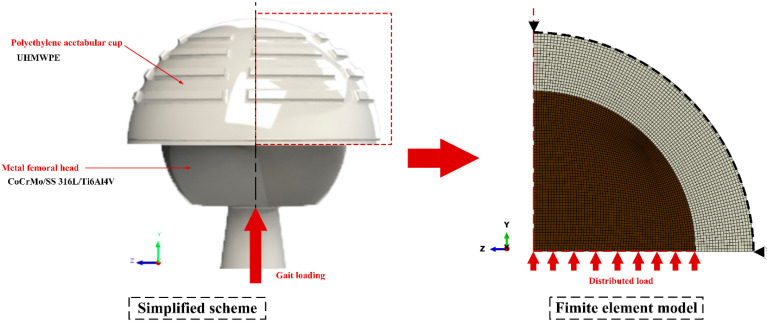
Simplified scheme and finite element model of Metal-on-UHMEPE couple bearing.

The femoral head and acetabular cup components were represented in the form of a two-dimensional axisymmetric finite element model in ABAQUS/CAE 6.14-1, shown in Figure 1, with 2000 CAX4 elements for the femoral head and 3500 CAX4 elements for the acetabular cup. The geometry of the model adopted the size of bearing suitable for the commonly Indonesian hip joint (28 mm femoral head diameter, 0.05 radial clearance, and 5 mm acetabular cup thickness) [22]. The fixation components, pelvic bone, and femoral stem were not included in the analysis process to make computations faster but still accurate because it does not significantly affect the computational simulation results obtained. The fixed constraint is created on the outer surface of the acetabular cup due to the fact this component does not move and attaches to the pelvic bone [23]. The force was applied to the symmetric axis of the femoral head.

### 2.2. Materials Properties

**Figure 2:**
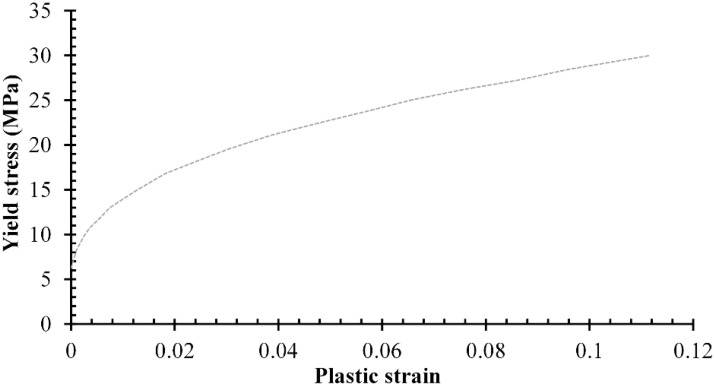
Plastic strain for UHMWPE acetabular cup [22].

Young’s modulus and Poisson’s ratio were used to define the mechanical properties of the investigated material for computational simulation needs, as presented in Table 1. All materials were assumed to be homogeneous and isotropic, but linear elastic for metals and non-linear plastic for UHMWPE. The definition of non-linear plastic in UHMWPE material for the acetabular cup component uses the relationship between plastic strain and yield stress described in Figure 2.

### 2.3. Coefficient of Friction

The asperity of contact interface between two bodies was defined by the coefficient of friction. This value was obtained from an experimental setup, either pin-on-disc [27] or hip joint simulator [28]. To represent asperity condition on bearing interface, the coefficient of friction is needed in computational simulation, provided in Table 2 for studied combination materials.

### 2.4. Gait Cycle

**Figure 3:**
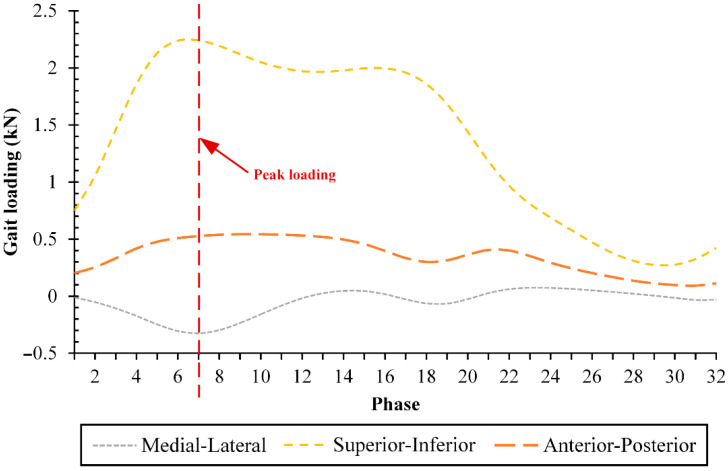
Triaxial forces under gait cycle [24].

One gait cycle was applied to the current computational model. The rationale for this is because most activities carried out by patients after hip joint replacement surgery are walking for the first time [29]. In adopting the gait cycle, the current study takes the magnitude of triaxial forces (medial–lateral, superior–inferior, anterior–posterior) as shown in Figure 3 from a previous study conducted by Jamari et al. [24] which provides a full gait cycle divided into 32 phases to simplify calculations, but without considering the range of motion as done by Basri et al. [30]. The largest resultant value was in the 7th phase of 2326 N, with superior–inferior forces dominating.

## 3. Results and Discussion

**Figure 4:**
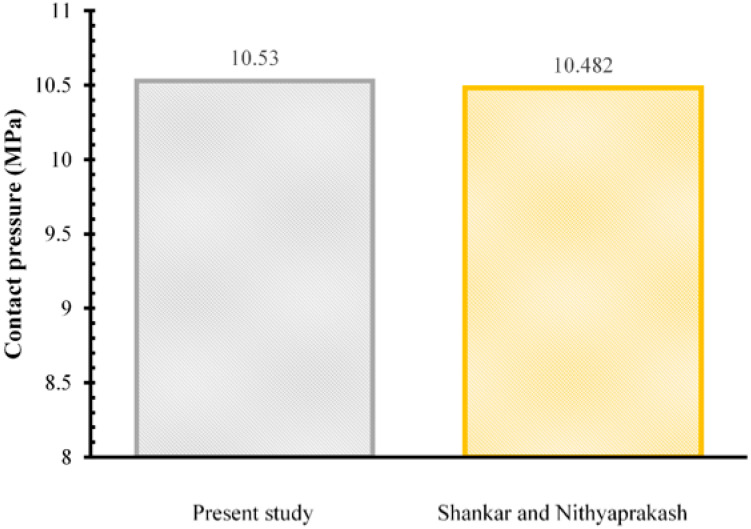
Contact pressure results comparison with Shankar and Nithyaprakash [21].

Results verification of the work with finite element computing is needed to ensure the validity of results obtained by comparing the results from published literature under similar conditions. For this purpose, the contact pressure result on CoCrMo-on-UHMWPE bearings in the 7th phase was verified with the results presented by Shankar and Nithyaprakash [21] shown in Figure 4. The difference in the contact pressure from current results with the literature is 0.048 MPa (4.58% difference from [21]). The percentage difference was below 10% so the current simulation results have been verified.

**Figure 5:**
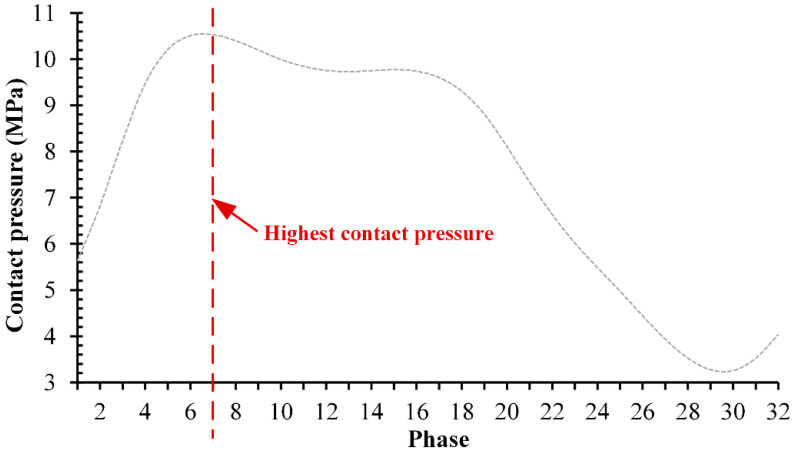
The maximum contact pressure of CoCrMo-on-UHMWPE from each phase under the gait cycle.

Figure 5 shows maximum contact pressure under full gait cycle for CoCrMo-on-UHMWPE as the representative of three different metal-on-UHMWPE. From the results obtained, it can be seen that from the initial phase the value of the contact pressure increases up to the highest in the 7th phase, then decreases until the lowest in the 30th phase until it finally rises slightly until the end of the gait cycle. The value of each phase changes due to the magnitude of the resultant force applied to provide conditions under the gait cycle.

The highest contact pressure value can be seen in Table 3. Contact pressure from the highest to the lowest were found in Ti6Al4V-on-UHMWPE, SS 316L-on-UHMWPE, and CoCrMo-on-UHMWPE, respectively. When compared with CoCrMo-on-UHMWPE as a combination of bearing material with the lowest contact pressure, during the 7th phase a decrease of 0.028 MPa (0.265%) with SS 316L-on-UHMWPE and 0.188 MPa (1.754%) with Ti6Al4V-on-UHMWPE was found. The difference in contact pressure between UHMWPE acetabular cup and the three different types of metal femoral heads is due to the material properties of each metallic material, namely Young’s modulus and Poisson’s ratio. However, because the Poisson’s ratio for all metallic materials is the same as 0.3, the property that has a role in the difference in contact pressure results is Young’s modulus.

**Figure 6:**
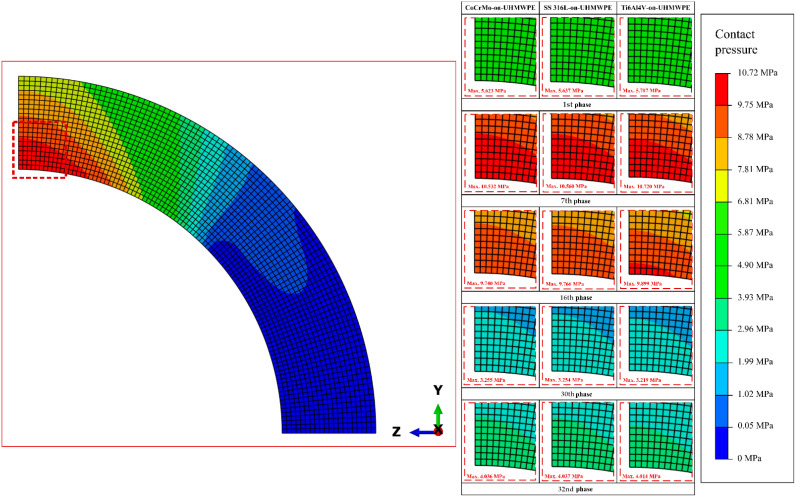
Distribution contour of contact pressure on UHMWPE acetabular cup.

Current simulation results obtained have shown contact pressure contours for the three types of metal-on-UHMWPE bearings in Figure 6. The contour is accessed using the post viewer from ABAQUS/CAE 16.4-1 on S, S22 menu [31]. To explain changes in the contact pressure contour, five phases were selected as representatives of the 32 phases under gait cycle, referring to previous research conducted by Ammarullah et al. [26]. It can be seen that the distribution of contact pressure will widen as the value of contact pressure and the applied force increase. Therefore, the 7th phase that is given the largest resultant force under gait cycle has the highest contact pressure and the widest contact pressure distribution compared to the other phases. The opposite is true in the 30th phase. Meanwhile, the area of highest contact pressure on the distribution contour is always in the centre of the contact area on the acetabular cup. The explanation of gait cycle loading in the current study does not adopt a range of motion. Thus, the force only works in the vertical direction.

**Figure 7:**
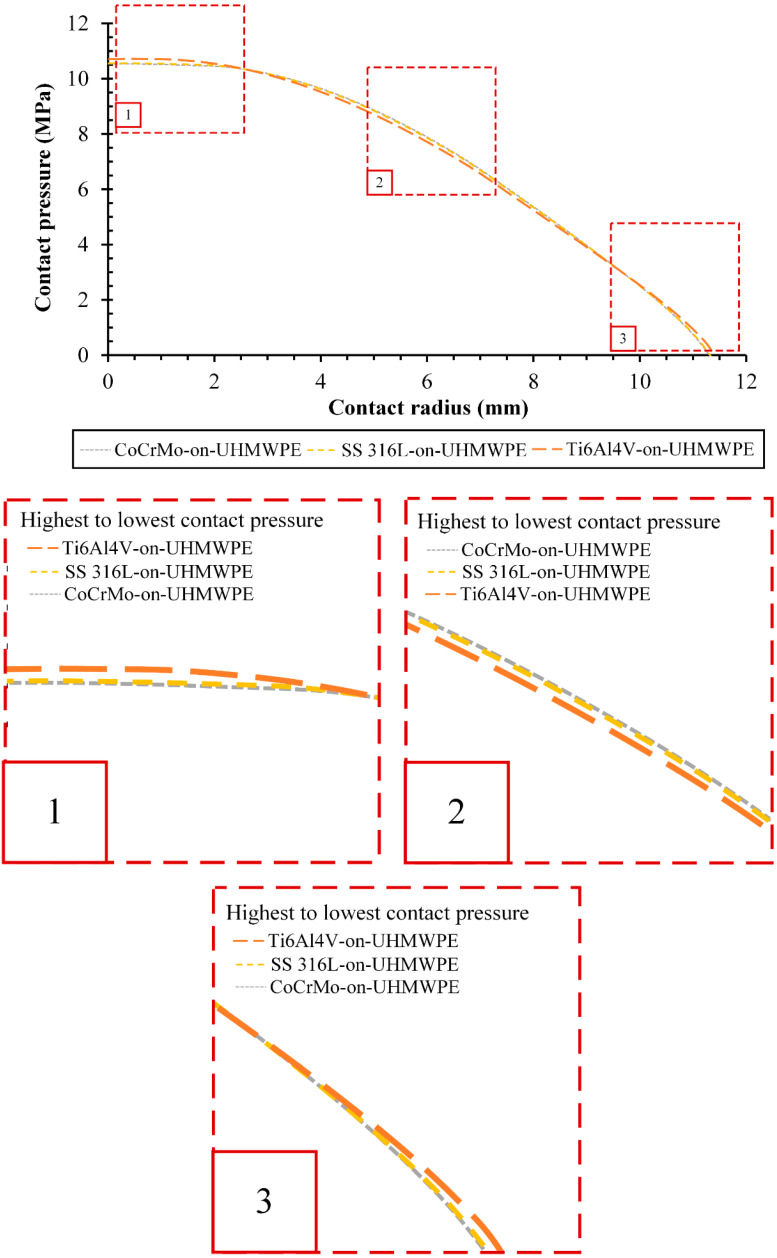
Correlation between contact pressure and contact radius during the 7th phase.

The distribution of contact pressure on the interface contact of UHMWPE acetabular cup in the 7th phase was studied by correlating contact pressure and contact radius described in Figure 7. Along with the distribution of contact pressure on UHMWPE acetabular cup, at the contact centre (see point number 1 in Figure 7) it can be seen that the highest contact pressure is experienced by Ti6Al4V-on-UHMWPE, followed by SS 316L-on-UHMWPE and CoCrMo-on-UHMWPE. Furthermore, at the middle contact radius (see point number 2 Figure 7) it can be seen that CoCrMo-on-UHMWPE has the highest contact pressure, followed by SS 316L-on-UHMWPE and Ti6Al4V-on-UHMWPE. At the end of contact (see point 3 Figure 7) it can be seen that the order of highest contact pressure returns to the same as a contact centre. Contact radius on the 7th phase for every material combination is shown in Table 4. This result is due to the soft characteristics of UHMWPE material in contact with harder metallic materials, indicated by the different values of Young’s modulus for UHMWPE and metallic materials.

Furthermore, the calculation of cumulative contact pressure at each node along the contact interface of the UHMWPE acetabular cup on current two-dimensional axisymmetric is presented in Table 5. Although the highest contact pressure of 10.720 MPa is in the 7th phase by Ti6Al4V-on-UHMWPE, it has the lowest cumulative contact pressure of 375.404 MPa relative to the other studied metal-on-UHMWPE bearings. Compared to Ti6Al4V-on-UHMWPE at peak loading, the difference in cumulative contact pressure to respective maximum contact pressure is 9.740% for SS 316L-on-UHMWPE and 11.038% for CoCrMo-on-UHMWPE.

Based on the Archard wear equation [17], contact pressure is an important aspect in predicting wear. Therefore, efforts to reduce the cumulative contact pressure that occurs are crucial to prolong the life of hip implants. In the investigation of material selection for the metallic femoral head to be a counterpart of UHMWPE acetabular cup, the selection of Ti6Al4V is the best option for reducing wear due to its lower cumulative contact pressure relative to the other materials under investigation. Although the difference in maximum contact pressure of CoCrMo, SS 316L, and Ti6Al4V is relatively small, this value will greatly affect the progress of wear rate, especially during the running-in wear phase since a slight increase of contact pressure greatly affects wear rate during this wear phase.

The discussion in terms of biocompatibility of metal materials for a metal femoral head as the counterpart of UHMWPE acetabular cup is also interesting to study. This is because the metal femoral head in metal-on-UHMWPE has the potential to cause poisoning for patients. In the previous contact pressure simulation results, the use of Ti6Al4V for the femoral head material provides the lowest cumulative contact pressure, meaning it has the lowest wear rate, but the choice of this material is also more promising from a biocompatibility perspective. According to the explanation of Ali et al. [32], compared to CoCrMo and SS 316L, Ti6Al4V has superior biocompatibility. This means Ti6Al4V can minimize the various possible negative biological responses for a patient during implant use, especially in the long term.

Apart from biocompatibility, the choice of Ti6Al4V is also supported by its excellent corrosion resistance property. Zaman et al. [33] have explained that compared to CoCrMo and SS 316L, Ti6Al4V has a better corrosion resistance property. Corrosion due to friction will lead to the release of metal ions which cause tissue reactions in the user’s body. The corrosion resistance property can minimize the release of metal ions from the implant surface when friction occurs.

The current study using bearing geometry of total hip arthroplasty focused on Indonesian body types (broadly applicable to Asian) with a femoral head diameter of 28 mm [34]. Unfortunately, the hip joint geometry of Asian people is different from other regions. In Europe, the femoral head tends to use a 32 mm diameter [35]. Europeans have a relatively larger size of hip joints than Asians. In further research, apart from studying the material selection aspect for ceramic materials that are not provided in the present manuscript, it is also necessary to study bearing geometry. The 28 mm diameter femoral head used by most Asians has a different behaviour compared to the 32 mm diameter femoral head used by most Europeans.

## 4. Conclusions

The current computational simulation successfully described the contact pressure evaluation of a metallic femoral head to become the counterpart of UHMWPE acetabular cup under the gait cycle. The choice of material is intended to reduce contact pressure since it is correlated with wear based on the Archard wear equation so that it can extend the life of total hip arthroplasty. Of the three types of combined components of the metal-on-UHMWPE bearings, it was found that the combination of UHMWPE acetabular cup and Ti6Al4V femoral head was the best choice to minimize cumulative contact pressure, indicating that it is able to reduce the wear rate compared to CoCrMo and SS 316L. The choice of Ti6Al4V as a material is also promising considering its superior biocompatibility and corrosion resistance aspect. For orthopaedists, the combination of Ti6Al4V femoral head with UHMWPE acetabular cup for total hip arthroplasty can be an option for material selection oriented towards developing countries, especially for Indonesian and mostly Asian people.

## Figures and Tables

**Table 1:** Young’s modulus and Poisson’s ratio for metal and UHMWPE simulated materials.

Component	Material	Young’s Modulus	Poisson’s Ratio	Reference
Femoral head	CoCrMo	210 GPa	0.3	[24]
	SS 316L	193 GPa		[25]
	Ti6Al4V	110 GPa		[26]
Acetabular cup	UHMWPE	1.4 GPa		[21]

**Table 2:** Coefficient of friction for different materials combination.

Material’s Component		Coefficient of Friction	Reference
Femoral Head	Acetabular Cup		
CoCrMo	UHMWPE	0.11	[22]
SS 316L	UHMWPE	0.1	[26]
Ti6Al4V	UHMWPE	0.0561	[26]

**Table 3:** Maximum contact pressure during the 7th phase.

Materials Combination	Contact Pressure
CoCrMo-on-UHMWPE	10.532 MPa
SS 316L-on-UHMWPE	10.560 MPa
Ti6Al4V-on-UHMWPE	10.720 MPa

**Table 4:** Contact radius on 7th phase.

Materials Combination	Contact Radius (mm)
CoCrMo-on-UHMWPE	7.686
SS 316L-on-UHMWPE	7.608
Ti6Al4V-on-UHMWPE	7.590

**Table 5:** Cumulative contact pressure analysis on 7th phase.

Materials Combination	Cumulative Contact Pressure (MPa)	Difference (MPa)	Comparison with Respective Maximum Contact Pressure (%)
CoCrMo-on-UHMWPE	376.566	1.162	11.038
SS 316L-on-UHMWPE	376.432	1.028	9.740
Ti6Al4V-on-UHMWPE	375.404	0	0

## Data Availability

The data presented in this study are available on request from the corresponding author.
